# Association between RNAm-Based COVID-19 Vaccines and Permanency of Menstrual Cycle Alterations in Spanish Women: A Cross-Sectional Study

**DOI:** 10.3390/biology11111579

**Published:** 2022-10-27

**Authors:** Isabel Mínguez-Esteban, Pablo García-Ginés, Carlos Romero-Morales, Vanesa Abuín-Porras, Jose A. Navia, Jose Luis Alonso-Pérez, Mónica de la Cueva-Reguera

**Affiliations:** 1 Faculty of Sport Sciences, Universidad Europea de Madrid, C/Tajo s/n, Villaviciosa de Odón, 28670 Madrid, Spain; 2 Facultad de Educación, Universidad de Alcalá, 28801 Madrid, Spain

**Keywords:** menstrual cycle alterations, vaccination, COVID, women

## Abstract

**Simple Summary:**

The purpose of this study was to delve more deeply into the medium and long-term relationship between mRNA-based vaccines and changes in menstrual pain, cycle length, and amount of bleeding in Spanish women. A total of 746 women (63% between 18–30 and 37% between 31–45 years old) participated in the study. Sixty-five per cent of the women perceived changes in their menstrual cycle after receiving the vaccines, irrespective of the type of vaccine or number of doses the novelty of the present study is that it confirms that menstrual cycle alterations in this sample could last more than 5 months after vaccination.

**Abstract:**

Introduction: The purpose of this study was to delve more deeply into the medium and long-term relation between mRNA-based vaccines and changes in menstrual pain, cycle length, and amount of bleeding in Spanish women. Material and Methods: A total of 746 women (63% between 18–30 and 37% between 31–45 years old) participated in the study. A numerical rating scale was used for recording pain intensity, a pictorial chart for menstrual bleeding, and data from menstrual cycle duration, type of vaccine, number of doses and time from vaccination. Results: Sixty-five per cent of the women perceived changes in their menstrual cycle after receiving the vaccines, irrespective of type of vaccine or number of doses; all *p* values were >0.05. Most of them (*n* = 316 out of 484) reported more than one alteration in their menstrual cycle. Almost half of the participants had been vaccinated over 5 months (45%), 3–4 months (15%) 2–3 months (26%), and one month or less (13%) before. The percentage of women that reported alterations remained strongly constant across time, *p* > 0.05, ranging from 64 to 65%. Conclusions: Reported alterations in Spanish women after COVID vaccination remained more than 5 months after the last dose.

## 1. Introduction

The COVID-19 disease, caused by the SARS-CoV-2 virus, has led to a series of unexpected adverse events, besides the common respiratory symptoms, such as central nervous system alterations or musculoskeletal disorders [1]. The pandemic has caused significant morbidity and mortality, as well as important social, educational and economic challenges [2]. Particularly, since 22 June 2020, 11,274,621 cases have been reported in Spain, with a prevalence in women of 52.8% (https://www.isciii.es (Accessed on 25 May 2022)). In such situations, vaccination has been demonstrated throughout history to be the most successful way to prevent infectious diseases from spreading [3]; effective, long-term vaccines are considered to be the main way to generate community immunity [4]. Authorized vaccines in Spain with mRNA technology (Pfizer-BioNTech and Moderna) have been shown to be highly effective against symptomatic COVID-19 infections. Moreover, the efficacy of full vaccination with two doses is estimated at 94% [5]. Some of the side effects of these vaccines may include: pain, redness, swelling, tiredness, headache, fever or nausea. In addition, thrombosis has been reported as a rare, severe side effect of vaccination [6].

Vital organ injuries caused by the COVID-19 disease are still partially unexplained. The lungs, heart, and kidneys seem to be specifically targeted by the virus [7]. A common factor in these organs is that they exhibit high levels of Angiotensin-Converting Enzyme-2 (ACE2), which is essentially the binding area in human cells for SARS-CoV-2 [8]. ACE2 is a carboxypeptidase that negatively regulates the Renin–Angiotensin System and provokes vasodilatation through the scission of ACE2 [9]. Moreover, this transcellular protein is mainly expressed in the ovaries, uterus, vagina and placenta [10]. ACE2 regulates follicle development and ovulation, modulates luteal activity, and induces endometrium changes. Several studies have hypothesized that the COVID-19 disease could induce alterations in female physiological functions through this existent relation between ACE2 and SARS-CoV-2 [11].

Alterations in the menstrual cycle (MC) have been quite a reiterative topic in recent scientific literature, and the search for possible causes for this condition is continuously evolving [12,13,14]. Normal MC occurs cyclically with a mean duration of 28 days. However, variations from 21 to 37 days are considered unimportant [15]. There are several menstrual disorders—dysmenorrhea, endometriosis, or premenstrual syndrome, amongst others—that can affect women in their fertile years [16,17,18]. Normal menstrual bleeding lasts from 2 to 7 days, and during this time, 50–120 mL of blood is lost. Hypermenorrhea is diagnosed if the amount of blood is greater than 120 mL, or the length of the cycle is beyond 7 days. If the quantity of eliminated blood is less than 50 mL or the cycle lasts less than 2 days, it is classified as hypomenorrhea [19]. In addition, another common alteration in MC is dysmenorrhea, defined as pain in the lower abdominal area occurring prior to or during menstruation, without any identifiable pelvic pathology [20].

Several studies conducted in Spain, UK, USA and Norway have compiled a wide range of alterations in MC related to COVID-19 [21]. A recent prevalence study conducted in Spain reported changes in MC right after vaccination [22]. The purpose of this study was to delve further into the medium and long-term relationship between the most used vaccines in Spain (Pfizer and Moderna, both mRNA based) and changes in menstrual pain, cycle length, and amount of bleeding in a sample of Spanish women.

## 2. Materials and Methods

This study follows an observational, descriptive, cross-sectional model, according to the STROBE [23] guidelines, where participants were requested to answer an online survey after accepting to sign an informed consent form. The study was advertised through social websites, and approved by the Ethics Committee from the Universidad Europea de Madrid (CPI/22.126). Obtained data have been processed following the 3/2018 law for Personal Data Protection and the declaration of Helsinki [24].

### 2.1. Participants

The total number of participants completing the online questionnaire and assessed for eligibility was 2485. After applying exclusion criteria, the final number of participants analyzed for the study was 746. Inclusion criteria were: (a) Women between 18 and 45 years; (b) Living in Spain; (c) with two doses of mRNA vaccine (Pfizer or Moderna) received; (d) Using a tampon, menstrual pad or cup. Exclusion criteria were: (a) Vaccinated with non-mRNA-based vaccines (Johnson&Johnson, Novavax or AstraZeneca); (b) Previous pathologies that could interfere with normal MC (myomas, gynecological cancer, endometriosis, polycystic ovaries or thyroid alterations); (c) Using contraceptive methods or sterilization techniques; (d) Intake of drugs that could alter the MC (acetylsalicylic acid, anti-depressives or antipsychotic medication) (e) Menopausal or pre-menarche.

### 2.2. Outcome Measures

An online survey was designed for data collection, combining validated questionnaires and some items that were created for the purpose of this study. Collected data included information related to MC both previous and post vaccination. The survey was launched between February to June 2022, when the third dose was administered in Spain.

Pain intensity during menstruation pre and post vaccination was assessed with the validated numerical rating scale (NRS), rating pain from 0 (no pain) to 10 (excruciating pain).

MC duration was reported in days, from 1 to 14.

Moreover, a pictorial chart [25] was used to establish the menstrual bleeding quantity, scoring from 1 (mild bleeding) to 20 (heavy bleeding). To determinate the total amount of bleeding, this scoring was multiplied by the number of tampons or pads used.

For the rest of the questionnaire, self-designed questions were implemented to recall data from; type of vaccine, number of doses and time from vaccination.

### 2.3. Statistical Analysis

The distribution of categorical variables was analyzed through the standardized residuals of proportion of responses (Z’s) and the chi squared test (χ^2^). For the quantitative variables (pain, duration and bleeding amount), visual inspection of residuals and normality tests revealed a non-normal distribution of variables. Therefore, non-parametric tests were employed; Wilcoxon for paired related observations (i.e., across time) and Mann–Whitney or Kruskall–Wallis for independent group comparisons (across groups) over the difference in time (post–pre). Effect sizes were expressed in r, with values of 0.10, 0.3, and 0.5 for small, moderate and large effects [26]. Rho Spearman’s test was used to analyze relationships among variables. The level of significance for all analyses was set at α = 0.05. IBM SPSS V.25 package (Armonk, NY, USA) was used for the statistical analysis.

## 3. Results

Participants ranged between 18 and 45 years of age: 63% between 18–30 and 37% between 31–45. In terms of period supplies, 31% of women combined tampon and pads, 14% only tampons, 22% only pads, 19% menstrual cups and 14% other combinations or methods during the menstruation.

Regarding type of vaccine, 70% received Pfizer and 30% Moderna in the first dose, 69% and 31% in the second dose (*n* = 24 women shifted). At the moment of data collection, 57% had not received the third dose, whereas of those who had, 22% received Pfizer and 21% Moderna. From those participants with three doses, *n* = 192 received the same vaccine across the three doses, whereas *n* = 132 varied. Sixty-five per cent of the women perceived changes in their menstrual cycle after being administered the vaccines, irrespective of the type of vaccine or number of doses; all *p* values > 0.05. Most of them (*n* = 316 out of 484) reported more than one alteration in their menstrual cycle (Table 1). Older participants more frequently reported alterations (77% of women between 31–45 years) than younger ones (60% of woman between 18–30, χ^2^ (1) = 11.18, Z = 3.3, *p* < 0.001.

Almost half of the participants had been vaccinated over 5 months (45%), with the rest 3–4 months (15%) 2–3 months (26%), and one month or less (13%) before. The percentage of women that reported alterations remained strongly constant across time, *p* > 0.05, ranging from 64 to 65% (Table 2). In other words, reported alterations remained over 5 months since the last dose.

Women more often reported an increase—rather than a reduction—in duration, bleeding and pain during their menstrual cycles (Table 2). A similar effect was observed in the full sample, finding a significant rise in these variables (Table 3). That is, the women in this study experienced greater pain and duration of the menstrual cycle and bleeding amount after the COVID vaccines. These changes across time in pain, duration or bleeding were not influenced by type of vaccine or number of doses. The rise in pain, duration and bleeding after the vaccines was similar across the participants, regardless of the time since the last dose, all *p* values > 0.005. Unlike pain and bleeding, women between 31–45 years old experienced a greater rise in duration of MC (0.44 ± 1.26 days) than younger women (0.09 ± 1.26 days), Z = 3.68, *p* < 0.001.

Moreover, a positive relationship among pain, duration and bleeding was found both before and after the COVID vaccination (Table 4). The longer the duration of the menstrual period, the greater the pain and bleeding. Furthermore, women that after the vaccines experienced greater durations and bleeding also felt increased pain, as revealed by a positive correlation in the change post-pre (Table 4).

## 4. Discussion

The purpose of this observational study was to explore the permanence of MC alterations in women after the administration of mRNA-based vaccines. The results support the presence of one or more MC alterations even 5 months after vaccination, irrespective of the type of vaccine or combination between them, number of doses or time since last dose.

Several studies have been conducted in various countries ever since the appearance of the first menstrual irregularity reports after COVID-19 vaccinations, some of them focusing on the differences in the responses of the male and female immunity system [22]. Minankshi et al. [27] pointed out the specific impact of inflammatory processes in the ovary, and the global sensitivity of the female reproductive system to endocrine factors. The topic of particularities of the female immune response to vaccines is not uncommon in scientific literature [28]. Suzuki et al. [29] studied the relationship between human papillomavirus (HPV) and changes in MC after a national Japanese vaccination campaign. Gong et al. [30] also studied HPV vaccination and its relationship with premature ovarian insufficiency; still, there is lack of specificity in clinical trials when addressing gender-mediated immune responses [27]. Specifically, regarding COVID-19 vaccination, it is plausible that the importance of ACE2 in MC events and the relation of this protein with SARS-CoV-2 [31] could be a link between the vaccines and MC alterations (9). Moreover, some studies have reported changes in MC after COVID-19 infection [32], supporting the idea of a link between this specific virus activity and changes in feminine menstrual regulation. Nevertheless, these adverse events were not reported originally by pharmaceutical companies, possibly due to disregarding gender bias in the original clinical trials [33].

Women in this study reported changes in the duration in their menstrual cycle (24% increased, 12% decreased, 10% absence), increased bleeding and pain. These results coincide with those from Baena-García et al., also in a sample of Spanish women [22]. In this study, authors recorded data immediately after the vaccination campaign in order to avoid recall bias, so there was no follow-up of the duration of the changes. The novelty of the present study is that it confirms that MC alterations in this sample could last over 5 months after vaccination. Edelman et al. [34], in a US cohort and Trogstad et al. in Norwegian woman [35], also found alterations in MC in their studies, but in both studies, they limited their findings to one to three months after vaccination. In the Elderman et al. study, changes were reported to be minimal and explainable by normal MC dysregulation and even by pandemic stress [36]. The regulation of MC is multifactorial, and doubtless psychological factors cannot be disregarded [37]. In addition, those of the women in the Elderman et al. study who received both doses in the same cycle experienced a larger increase in their cycle duration than other participants. That dosage would be comparable to the normal Spanish prescription for mRNA vaccines.

Some of the results are challenging to compare with present literature, since dosage application varies depending on the country. Dosage in the UK has been reported to be 8 weeks apart, so the results of studies reporting changes in the length of CM in UK women are hardly comparable to those of the present study as mRNA vaccines are administered with a 21–28-day period between doses, meaning that some women are receiving both doses in one MC. However, the results also coincide, reporting an increase in bleeding and menstrual changes [21].

Regarding standardization of a data-recording protocol, the present study is, to the authors’ knowledge, the first in the scientific literature using questionnaires for the recording of pain symptoms [38] and menstrual blood amount [25]. This would strengthen the validity of our results, as self-designed surveys, though needed when a new situation arises, lack specificity. Several authors record changes in pain or bleeding amount in their studies, but the items in their questionnaires are dependent on the relative perception of the participants about changes in their pain or their bleeding [31,35,39].

### Limitations and Future Lines

As the outcomes were self-reported, the possibility of perception and recall biases must be pointed out. Moreover, the recruitment of the participants was carried out by advertising in social websites, so it is probable that women that did experience changes in their MC were more inclined to participate due to the general anxiety and constant broadcasting by the media about this kind of alteration during the vaccination campaign. Finally, participants were not asked about their expectations regarding changes to their menses. Expectation could plausibly act as a confounder regarding perception. Further studies with larger samples could include information about expectations in their data collection, as well as more specific sociodemographic data.

## 5. Conclusions

The results of this study support the existence of a correlation between mRNA vaccine administration and pain, duration of the menstrual cycle and bleeding amount disregarding type of vaccine, number of doses or time since last dose. Finally, a correlation between pain, duration and bleeding was found both before and after COVID19 vaccination. Public Health services should consider these data in order to discuss the inclusion of this information in their vaccination campaigns, to adjust population expectations regarding adverse events and moderate social concerns if alterations in MC appear.

## Figures and Tables

**Table 1:** Frequency and percentage of woman that reported alterations by time since last dose.

	Time Since Last Dose (Months)				
Alterations	0–1	2–3	3–4	>5	Total
Yes	65	125	75	219	484
No	34	71	39	117	261
Total	99	196	114	336	745
%	66	64	66	65	65

**Table 2:** Alterations regarding menstrual cycle reported after COVID vaccination.

	Counts	% of Total
↑ Duration of menstrual cycle	221	24
↓ Duration of menstrual cycle	113	12
↑ Bleeding amount	155	17
↓ Bleeding amount	85	9
↑ Pain	223	24
↓ Pain	27	3
Absence of menstrual cycle	95	10
Total	919	100

Note: total frequency is greater than sample size (*n* = 484) since 316 participants reported more than one alteration. ↑ Increase ↓ Decrease.

**Table 3:** Number (N), Mean (M), Inferior (I) and Superior (S) Confidence Interval of the Mean (95%), Standard Deviation (SD) of pain, duration and bleeding and contrast by moment (pre vs. post).

				95% CI of M			Wilcoxon		
		N	M	I	S	SD	Z	*p*	r
Pain	Pre	745	5.09	4.91	5.27	2.54	−9.80	<0.001	−0.36
	Post	745	5.79	5.60	5.98	2.64			
Duration	Pre	745	4.70	4.62	4.78	1.09	−5.15	<0.001	−0.19
	Post	745	4.92	4.83	5.01	1.25			
Bleeding	Pre	675	105.64	100.27	111.01	71.07	−2.77	0.006	−0.11
	Post	664	110.45	104.81	116.09	74.00			

Note. For bleeding amount, some participants did not respond (*n* < 745).

**Table 4:** Spearman’s Rho correlation before menstrual pain, duration and bleeding and after the vaccines.

	Pre		Post		Change (Post-Pre)	
	Pain	Duration	Pain	Duration	Pain	Duration
Duration	0.16 ***		0.15 ***		0.34 ***	
Bleeding	0.19 ***	0.32 ***	0.18 ***	0.32 ***	0.32 ***	0.47 ***

Note. *** *p* < 0.001.

## Data Availability

Under reasonable request.
